# Reviewed article: Rozman LM, Sartori AMC, Souza AA, Soarez PC. Discrepancy between official records of the Brazilian National Immunization Program Information System and the household survey about vaccination coverage in Cubatão, São Paulo: cross-sectional study, 2023. Epidemiol Serv Saude. 2025:34;e20240523

**DOI:** 10.1590/S2237-96222025v34e20240523.b

**Published:** 2025-08-11

**Authors:** Angélica Yukari Takemoto

**Affiliations:** Centro Universitário Guairacá

**Table:** 

Rating	Excellent	Good	Average	Below average	Poor
Interest		✓			
Quality		✓			
Originality	✓				
Overall		✓			

**Table:** 

Questionnaire	Yes	No	Not applicable
Does the manuscript contain new and significant information to justify publication?	✓		
Does the Abstract (Summary) clearly and accurately describe the content of the article?	✓		
Is the problem significant and concisely stated?	✓		
Are the methods described comprehensively?		✓	
Are the interpretations and conclusions justified by the results?	✓		
Is adequate reference made to other work in the field?		✓	

## Manuscript Structure

Length of article is: Adequate

Number of tables is: Adequate

Number of figures is: Adequate

Please state any conflict(s) of interest that you have in relation to the review of this paper (state “none” if this is not applicable): None

## Comments

O artigo intitulado “DESCOMPASSO ENTRE REGISTROS OFICIAIS DO SISTEMA DE INFORMAÇÃO DO PROGRAMA NACIONAL DE IMUNIZAÇÕES E INQUÉRITO DOMICILIAR DE COBERTURA VACINAL EM CUBATÃO, SÃO PAULO, 2023” foi avaliado e sugere-se a publicação, porém, com ajustes a serem realizados pelos autores. O estudo está bem escrito e delineado, concordando com os objetivos propostos. Acredita-se que os resultados do presente estudo auxiliarão em melhorias nos registros das imunizações entre os menores de dois anos, principalmente no que se refere ao sistema do PNI. 

